# The role of transient receptor potential melastatin channels in compressive force‐induced contraction of primary cardiac pericytes

**DOI:** 10.14814/phy2.70396

**Published:** 2025-06-17

**Authors:** Carmen Methner, Eugene Cilento, Zhiping Cao, Jeffrey Iliff, Anusha Mishra, Sanjiv Kaul

**Affiliations:** ^1^ Knight Cardiovascular Institute Oregon Health & Science University Portland Oregon USA; ^2^ Department of Anesthesiology & Perioperative Medicine Oregon Health & Science University Portland Oregon USA; ^3^ Jungers Center for Neurosciences Research, Department of Neurology Oregon Health & Science University Portland Oregon USA

**Keywords:** compressive forces, myocardial infarction, TRPM channel

## Abstract

Pericytes contract during acute myocardial infarction (AMI) resulting in capillary constriction, which further contributes to the ischemic damage and enlargement of infarct size. We hypothesized that increased intramyocardial pressure during ischemia can be sensed by mechanosensitive Transient Receptor Potential (TRP) channels in cardiac pericytes, resulting in their contraction and worsening of myocardial necrosis during AMI. Here, we show that cultured primary cardiac pericytes express several TRP channels. Live‐cell confocal imaging demonstrates that pharmacological stimulation with specific TRPM4 and TRPM7 agonists mobilizes cytosolic Ca^2+^ within pericytes. Pressure stimulation (increased compressive forces) also increases pericyte Ca^2+^, which is abolished by specific TRPM4 and TRPM7 inhibitors. Lastly, we demonstrate that TRPM4 inhibition in vivo reduces infarct size by 3.5‐fold in a rodent AMI model. We conclude that pericytes sense increased compressive forces (pressure) via TRPM channels both in vitro and in vivo. Inhibiting TRP channels may offer a therapeutic option to reduce infarct size in patients experiencing AMI.

## 1 INTRODUCTION

Myocardial ischemia causes regional myocardial dysfunction varying from hypokinesia to dyskinesia (Kaul, 1990; Lieberman et al., 1981). This regional dysfunction is associated with increased intramyocardial compressive forces during systole (Avazmohammadi et al., 2020; Downey et al., 1979). We, therefore, hypothesized that increased intramyocardial pressure during ischemia can be sensed by pericytes resulting in their contraction. Transient receptor potential melastatin (TRPM) channels have been reported to act as mechanosensors in various tissues, including the cardiovascular system (Earley, 2013; Inoue et al., 2009; Starostina et al., 2021). They are reported to be present in brain pericytes (Hariharan et al., 2020). We recently also identified them in mouse primary cardiac pericytes (Cao et al., 2022). In this study, we hypothesized that TRPM channels present in pericytes act as mechanosensors, initiate pericyte Ca^2+^ mobilization, and lead to their contraction.

We used live‐cell confocal imaging of cultured primary cardiac pericytes to demonstrate that pressure stimulation mobilizes Ca^2+^ within pericytes via the mechanosensitive receptors TRPM4 and TRPM7. It was previously reported that pre‐treatment with the TRPM7 inhibitor, carvacol, reduces infarct size in a rodent model (Yu et al., 2013). Here we show that in vivo inhibition of TRPM4 after coronary occlusion also reduces infarct size in wild‐type mice, likely by inhibiting pericyte contraction and allowing adequate capillary flow.

## 2 METHODS

All animal procedures performed in this study were approved by the Institutional Animal Care and Use Committee of Oregon Health & Science University and adhered to NIH Guide for the Care and Use of Laboratory Animals. Animals were housed in groups in the rodent vivarium and were fed regular chow (PicoLab Rodent Diet 5LOD by Purina Mills, Inc.) ad libitum. Mice undergoing cell isolation or immunohistochemistry (IHC) were euthanized using isoflurane and cervical dislocation. For cell isolation, mice from Jackson Laboratories were used, and for IHC, NG2‐DsRed mice (Methner et al., 2019; Mishra et al., 2014; Zhu et al., 2008) were used.

### 2.1 Isolation and culture of pericytes from adult mouse heart

Primary cardiac pericytes were isolated and cultured from mouse hearts as previously described (Cao et al., 2022). Briefly, ventricles were dissected from five 3–7‐weekweek‐old C57BL/6 male mice (Jackson Laboratories, Bar Harbor, ME), and then diced and digested with collagenase (CLS‐2, Worthington, Cat. #LS004176) in an agitated water bath (37°, 100 RPM) for 45 min. To isolate pericytes, single‐cell suspensions were plated on collagen‐I coated T‐75 culture flasks. Once confluent, the cells were sorted with 3G5‐conjugated Dynabeads (Thermo Fisher Scientific, Cat. #11039D). The Dynabead‐bound cells were then resuspended, plated in a collagen‐coated T75 flask, and grown until confluence.

### 2.2 Immunocytochemistry

Immunocytochemistry of pericytes was performed as previously described (Cao et al., 2022). Pericytes, cultured on glass coverslips, were fixed for 20 min in fresh 4% paraformaldehyde in phosphate buffered saline (PBS, 0.1 M sodium phosphate buffer, 0.9% NaCl, pH 7.4) and subsequently blocked with 4% normal goat serum (NGS, Millipore Sigma, Cat. #S26), 1% bovine serum albumin (BSA), and 0.3% Triton X‐100 in PBS for 45 min, then incubated overnight at 4°C with primary antibodies diluted in the blocking buffer. The following primary antibodies and dilutions were used: mouse anti‐α‐smooth muscle actin (α‐SMA), 1:200 (Sigma, Cat. #A2547); rabbit anti‐vimentin, 1:250 (Abcam, Cat. # ab92547); rabbit anti‐CD‐146, 1:100 (Proteintech, Cat. #17564‐1‐ap); rabbit anti‐platelet‐derived growth factor β (PDGFβ), 1:100 (Abcam, Cat. #ab32570); and rabbit anti‐neural‐glial 2 (NG2) chondroitin sulfate proteoglycan, 1:100 (Millipore Sigma, Cat. #AB5320). Cells were then rinsed 3 times for 10 min each in PBS and incubated with secondary antibodies conjugated with appropriate fluorophores for 1 h: Alexa Fluor 488 donkey anti‐rabbit IgG, 1:200 (Thermo Fisher Scientific, Cat. #A21206) or Alexa Fluor 488 goat anti‐mouse IgG, 1:200 (Thermo Fisher Scientific, Cat. #A11029). Cells were again rinsed 3 times for 10 min each in PBS and mounted on slides. The third rinse contained Phalloidin‐Alexa Fluor‐568 (Thermo Fisher Cat #A12380) and Hoechst 33342 (Thermo Fisher Cat. #62249) to label F‐actin and DNA, respectively. Images were captured using an inverted Nikon 1AR+ Resonant Scanning Confocal System. Images were analyzed using Image J (National Institutes of Health, Bethesda, MD).

### 2.3 Immunohistochemistry (IHC)

For IHC, NG2‐DsRed mouse heart ventricles were fixed with 4% paraformaldehyde. Frozen sections were cut at 20 μm thickness and washed with PBS, blocked in 10% NGS and 0.3% Triton X‐100 in PBS for 90 min, and then stained with primary antibodies overnight at 4°C: rabbit anti‐TRPM4, 1:100 (Alomone Labs, Cat. #ACC‐044) and rabbit anti‐TRPM7, 1:100 (Alomone Labs, Cat. #ACC‐047). After washing 3 times in PBS, sections were incubated with secondary antibodies for 2 h at room temperature: Alexa Fluor 488 donkey anti‐rabbit IgG, 1:200 (Thermo Fisher Scientific, Cat. #A21206) or Alexa Fluor 647 donkey anti‐rabbit IgG, 1:200 (Thermo Fisher Scientific, Cat. #A32795). Then, sections were washed 3 times in PBS and placed in an autofluorescence‐reduction solution (10 mM CuSO_4_, 50 mM ammonium acetate) for 30 min, and mounted on slides with SlowFade Gold Antifade Mountant (Thermo Fisher Scientific, Cat. #S36936) with 4′,6‐diamidino‐2‐phenylindole (DAPI, Invitrogen, Cat. #H3570). The sections were imaged with a Nikon 1AR+ Resonant Scanning Confocal System. Negative controls included sections in which the primary antibodies were omitted, incubated with secondary antibodies only, or sections in which both primary and secondary antibodies were omitted.

### 2.4 Western blot

Pericytes were lysed in lysis buffer (50 mM Tris, pH 7.4), 150 mM NaCl, 1% NP‐40, 0.25% Na‐deoxycholate, 1 mM ethylenediaminetetraacetic acid, and protease inhibitor (Millipore Sigma, Cat. #4693159001). The proteins prepared in sodium dodecyl sulfate (SDS) sample buffer (2% SDS, 10% glycerol, 80 mM Tris, pH 6.8,0.15 M β‐mercaptoethanol, 0.02% bromphenol blue) were separated on 4%–12% SDS–polyacrylamide gels and transferred to 0.45 μm polyvinylidene difluoride (PVDF) membranes (LI‐COR Cat. # 926‐31099). After completion of the transfer, blots were blocked in tris‐buffered saline (TBS) Odyssey Blocking Buffer (LI‐COR. Cat. # 927‐60001) for 1 h with gentle rocking at room temperature, incubated overnight (12–16 h with rocking) in primary antibody solution (rabbit anti‐TRPM4, 1:200 (Alomone Labs, Cat. #ACC‐044) and rabbit anti‐TRPM7, 1:200 (Alomone Labs, Cat. #ACC‐047) in 5% BSA in TBS with 0.1% Tween® 20 detergent [TBST]) at 4°C, and then incubated in secondary antibody solution (with 5% BSA in TBST, goat anti‐rabbit IgG IRDye 800, 1:10,000 (Licor Bioscience, Cat. #926‐32211)) for 1 h at room temperature. Finally, blots were imaged on an Odyssey Clx Imaging System (LI‐COR) and quantified using Image Studio software.

### 2.5 RNA sequencing

The RNA sequencing (RNAseq) profiling was obtained from a published database from our laboratory (Cao et al., 2022). The method of RNA preparation and RNAseq analysis was as follows: RNA was extracted from confluent cultures of pericytes (1 T75 flask/sample) using TRIzol (Invitrogen, Cat. #15596026) followed by the RNAeasy universal mini RNA kit according to each manufacturer's instructions. PolyA(+) RNA was next isolated using oligo‐dT bound to magnetic beads, and the recovered RNA was chemically fragmented to about 200–300 bp. Random hexamer priming was then used to produce double‐stranded cDNA, and proprietary adaptors (Illumina) were attached to the fragments by ligation.

The library is amplified by limited rounds of polymerase chain reaction (PCR). The amplified library was cleaned using AMPure XP beads (Agencourt Bioscience) and then run on the Bioanalyzer (Agilent Technologies) to ensure successful library preparation. HiSeq 2000 (Illumina) was used to sequence 100‐cycle single reads, and resulting data were converted to fastq files with CASAVA (Illumina). Subsequent trimming of known adapters and low‐quality regions of reads was performed using Fastq‐mcf, and reads were assigned to genes using the program “featureCounts”, part of the Subread R package. Reads that were aligned uniquely to the reference sequence were used for further analyses. Tags with <0.5 cpm in at least 2 samples were excluded. TMM normalization was performed with edgeR, and log2cpm values were calculated.

### 2.6 Quantitative PCR

Total RNA was isolated from pericytes using either the *mir*Vana PARIS RNA or Protein Purification Kit (Thermo Fisher Scientific, Cat. #AM1556) or the RNeasy Plus Mini Kit (Qiagen, Cat. #74104). RNA quality was inspected and verified on a NanoDrop prior to further use. Approximately 1 μg of RNA was reverse‐transcribed to generate first‐strand complementary DNA (cDNA) with a high‐capacity RNA‐to‐cDNA Kit (Thermo Fisher Scientific, Cat. #4387406). cDNA was subjected to PCR amplification using gene‐specific Taqman primers (Thermo Fisher Scientific). The 20 μL reaction contained Taqman Universal Master Mix II, no UNG (Thermo Fisher Scientific, Cat. #4440040), Taqman primers (Thermo Fisher Scientific, TRPM4: Mn00613173_m1, TRPM7: Mn00457998_m1, β‐Actin: Mn02619580_g1, TRPC1: Mn00441975_m1, TRPC2: Mn01274200_g1), RNA, and nuclease‐free H_2_O. The experiments were performed by following Taqman's instructions with the Applied Biosystems ViiA™ 7 Real‐Time PCR System.

### 2.7 Ca^2+^ imaging and compressive force assay

Cells were plated on a series of 35 mm dishes with 14 mm micro‐wells (Cellvis; Cat. # D35‐14‐1.5‐N) 48–96 h prior to experimentation and allowed to grow to 80%–90% confluence. On the day of imaging, cells were rinsed with PBS, loaded with the cell permeant Ca^2+^ dye Fluo‐4 AM (Thermo Fisher Scientific, Cat. #F14201) diluted to 20 μM in Hanks' Balanced Salt Solution (HBSS^+/+^) containing free Ca^2+^ and Mg^2+^ (Gibco, Thermo Fisher Scientific, Cat. #14025‐092) and F‐12, and incubated for 30 min at 37°C + 5% CO_2_. Finally, cells were rinsed with HBSS^+/+^ and submerged in 4 mL HBSS^+/+^ prior to imaging. All imaging was performed with an inverted Nikon 1AR+ Resonant Scanning Confocal System using an ultra‐speed resonant scanner and 20X air immersion objective. Images were captured every 500 ms with 4X averaging. Pharmacological agents were applied by pipette injection away from the imaging plane and mixed by gentle pipetting of the final solution. Prior to applying compressive force, a glass coverslip was gently placed atop the monolayer of cells followed by a 2–5 min delay to account for any subtle activity induced by coverslip application.

Calibration weights (Learning Resources, Cat. #LER 265) were gently placed atop the glass slip covering the Fluo‐4 AM loaded cells (Kanzaki et al., 2002). A series of weights ranging from 0.1 g to 2 g were tested in stepwise increments, and the 1 g weight was determined to induce the maximal Ca^2+^ mobilization without visibly tearing the cells apart or off the dish (data not shown). Therefore, the 1 g calibration weight, applying 1 g/cm^2^ total force, was chosen.

### 2.8 In‐vivo acute myocardial infarction experiments

In‐vivo experiments were performed to block TRPM4 in a rodent acute myocardial infarction (AMI) model (Methner et al., 2021). Fifteen wild‐type C57BL/6 mice (12–24 weeks old) of both sexes were used for the experiment for control (*n* = 8, 4M/4F) and intervention (*n* = 7, 3M/4F) groups. Anesthesia was induced and maintained with Isoflurane (3% induction, 1.5% maintenance). Electrodes were placed on both forelimbs and the right hind limb for continuous electrocardiographic monitoring. The thorax was opened in the 4th intercostal space, enlarged by cutting the intercostal muscles and retracted gently to allow access to the heart. The pericardium was opened and the left coronary artery (LCA) identified.

An 8–0 ligature was placed around the LCA, PE‐10 tubing was threaded through the ligature suture and held in place with a clamp to occlude the artery. Fifteen minutes later, 100 μL of either drug or vehicle was administered intravenously in a random order. The specific TRPM4 inhibitor, 9‐phenanthrol (1 mg/kg, Millipore Sigma, Cat. #648492) diluted in vehicle (saline:DMSO:Tween‐80 = 97.75:1.25:1) was used in the drug group. After 45 min of ischemia, the PE tubing was gently removed to reperfuse the heart for 2 h.

To quantify the risk area, the LCA was briefly re‐occluded at the end of reperfusion, and Evans Blue dye was injected intravenously (Methner et al., 2021). The heart was then excised, frozen for 10 min, sliced along the short‐axis, and stained with triphenyltetrazolium chloride (TTC; 1% in PBS, Fisher Scientific, Cat. #AAA1087018) for 20 min at 37°C, followed by incubation in 10% neutral buffered formalin overnight. Heart sections were then imaged using a digital camera to quantify infarct size as the area of tissue lacking TTC staining, which was then normalized to the risk area (Methner et al., 2021).

### 2.9 Statistical methods

Unless otherwise noted, data are expressed as mean ± 1 standard deviation. Differences were considered statistically significant at *p* < 0.05. In vitro data were compared using analysis of variance (ANOVA) followed by Kolmogorov–Smirnov post hoc test. Infarct size/risk area data were evaluated using unpaired nonparametric Mann–Whitney test.

## 3 RESULTS

### 3.1 Characterization of primary cardiac pericytes

**FIGURE 1:**
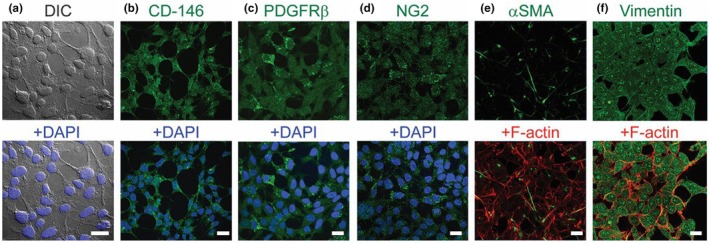
Confocal immunocytochemistry of primary cardiac pericytes. (a) Primary cardiac pericytes exhibit a single, uniform morphology in culture as demonstrated by confocal differential interference contrast imaging (gray). Primary cardiac pericytes also maintain expression of pericyte markers CD‐146 (b), PDGFRβ (c), NG2 (d), α‐SMA (e), and Vimentin (f) shown by immunocytochemistry overlaid with F‐actin marker Phalloidin (red). Images in the bottom panel show Hoechst 33342 staining for nuclei. Scale bar = 20 μm.

Differential interference contrast (DIC) microscopy of sub‐confluent cells confirmed a population of cells with a homogeneous morphology (Figure 1a). Immunocytochemistry demonstrated expression of known pericyte markers CD‐146 (Figure 1b), PDGFRβ (Figure 1c), NG2 (Figure 1d), α‐SMA (Figure 1e) and vimentin (Figure 1f). NG2 and PDGFRβ expression was found both diffusely throughout the cytoplasm as well as in clumps of perinuclear puncta, as demonstrated by their localization around DAPI staining (bottom panels Figure 1c,d). Expression of the intermediate filament and pericyte marker vimentin overlaps with F‐actin filaments (labeled with phalloidin), while expression of the mural cell contractility marker smooth muscle actin (α‐SMA) does not overlap (Figure 1e). Finally, as shown previously by us (Cao et al., 2022), pericytes were negative for markers specific to other vascular cell types, such as the vascular smooth muscle cell (VSMC) marker smooth muscle myosin heavy chain and epithelial cell marker CD31.

### 3.2 Mechanosensitive ion channels in pericytes

**FIGURE 2:**
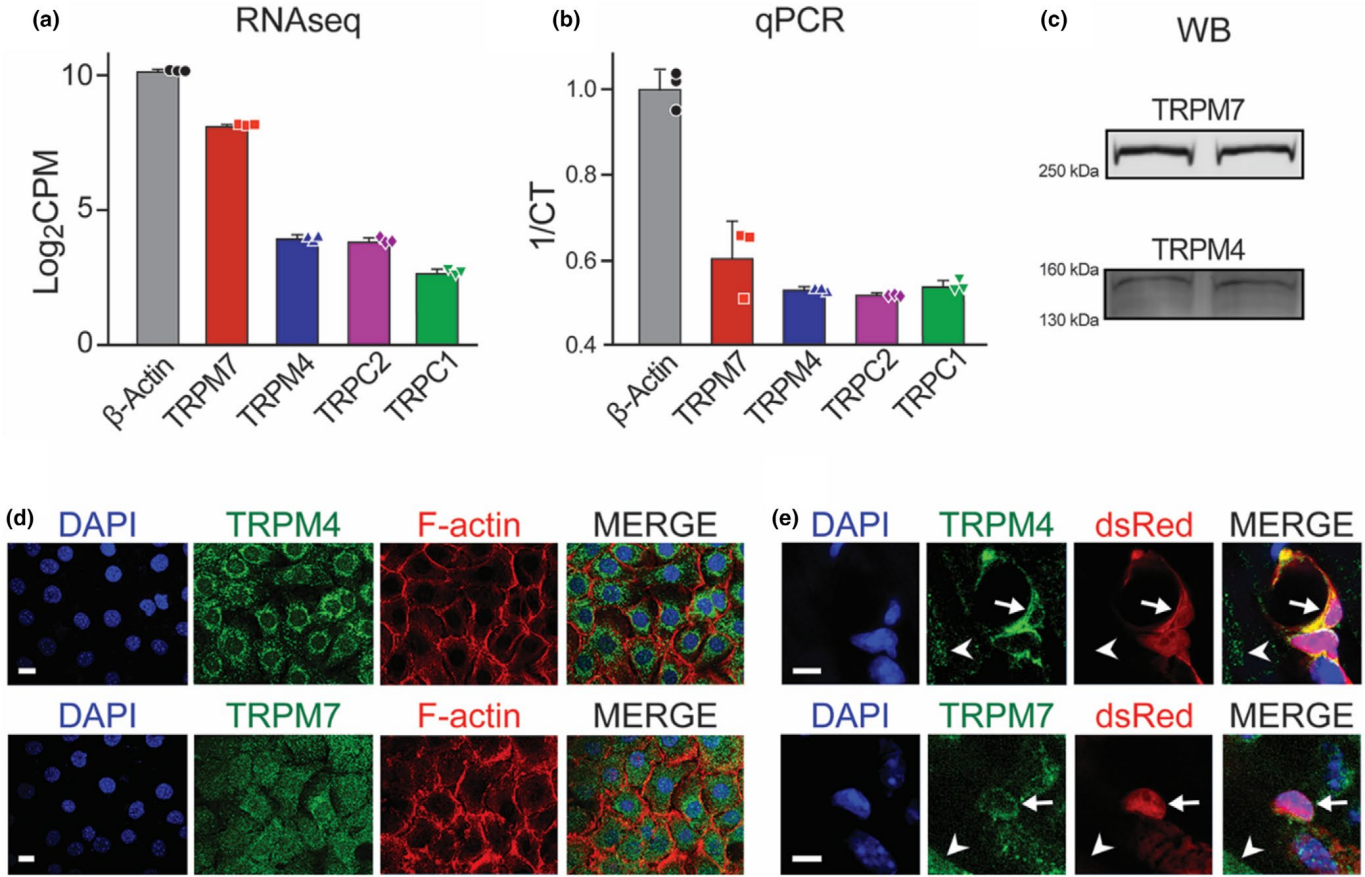
TRPM4 and TRPM7 are expressed in primary cardiac pericytes. (a) Global RNAseq from 3 separate cultures identified four TRP channels present in primary cardiac pericytes: TRPM7, TRPM4, TRPC2, and TRPC1. Expression is plotted as a function of detected Log_2_CPM relative to β‐actin. (b) Relative mRNA abundance was validated by quantitative PCR, with TRPM7 and TRPM4 again detected at highest abundance. Expression is plotted as a function of 1/cycle threshold (CT) relative to β‐actin. (c) Immunoblotting cellular lysates of primary cardiac pericytes demonstrates protein expression of TRPM7 and TRPM4. (d) Immunocytochemistry demonstrates TRPM4 and TRPM7 expression. TRPM4 appears to strongly localize in perinuclear networks, while TRPM7 appears diffusely expressed throughout the membrane. Scale bar = 20 μm. (e) IHC of heart slices from transgenic mice expressing DsRed under the pericyte‐specific NG2 promoter demonstrates TRPM4 and TRPM7 expression in the coronary vasculature of mice. Both TRPM4 and TRPM7 are colocalized in DsRed positive cells with the size and morphological characteristics consistent with pericytes (arrows). Some expression is also observed in non‐vascular cells (arrowheads). Scale bar = 5 μm.

To identify potential mechanosensors endogenous to pericytes, RNAseq analysis was performed on three separate cultures. Within these results, we chose to focus our analysis on TRP channels given their high abundance and integral role in mediating Ca^2+^‐dependent stretch/pressure responses within vascular cells (Earley, 2013; Hariharan et al., 2020; Inoue et al., 2009; Starostina et al., 2021). Four TRP channels (TRPM7, TRPM4, TRPC2, and TRPC1) were identified by RNAseq in the three separate cultures. Figure 2a illustrates the log_2_ counts per million reads (log_2_CPMs) of each of these channels with β‐actin serving as a reference. Quantitative qPCR confirmed the presence and expression profile of these four TRP channels with their 1/cycle threshold (CT) relative to β‐actin (Figure 2b). Immunoblotting further confirmed protein expression of TRPM7 and TRPM4 in cellular lysates of cultured primary cardiac pericytes (Figure 2c).

Immunofluorescence demonstrated strong membrane and perinuclear localization of TRPM4 (Figure 2d, top panel) and TRPM7 (Figure 2d, bottom panel). To confirm endogenous expression of TRPM4 and TRPM7 within cardiac pericytes, IHC was performed on sections of NG2‐DsRed mouse heart (Figure 2e). Representative images demonstrate TRPM4 (top panel) and TRPM7 (bottom panel) expression of cells consistent with pericytes: DsRed positive (NG2 expressing) cells surrounding vessels < 10 μm. Together, these data suggest that myocardial pericytes endogenously express TRPM4 and TRPM7 and maintain RNA and protein expression of these channels.

### 3.3 TRPM4 and TRPM7 agonists induce Ca^2+^ mobilization in pericytes

**FIGURE 3:**
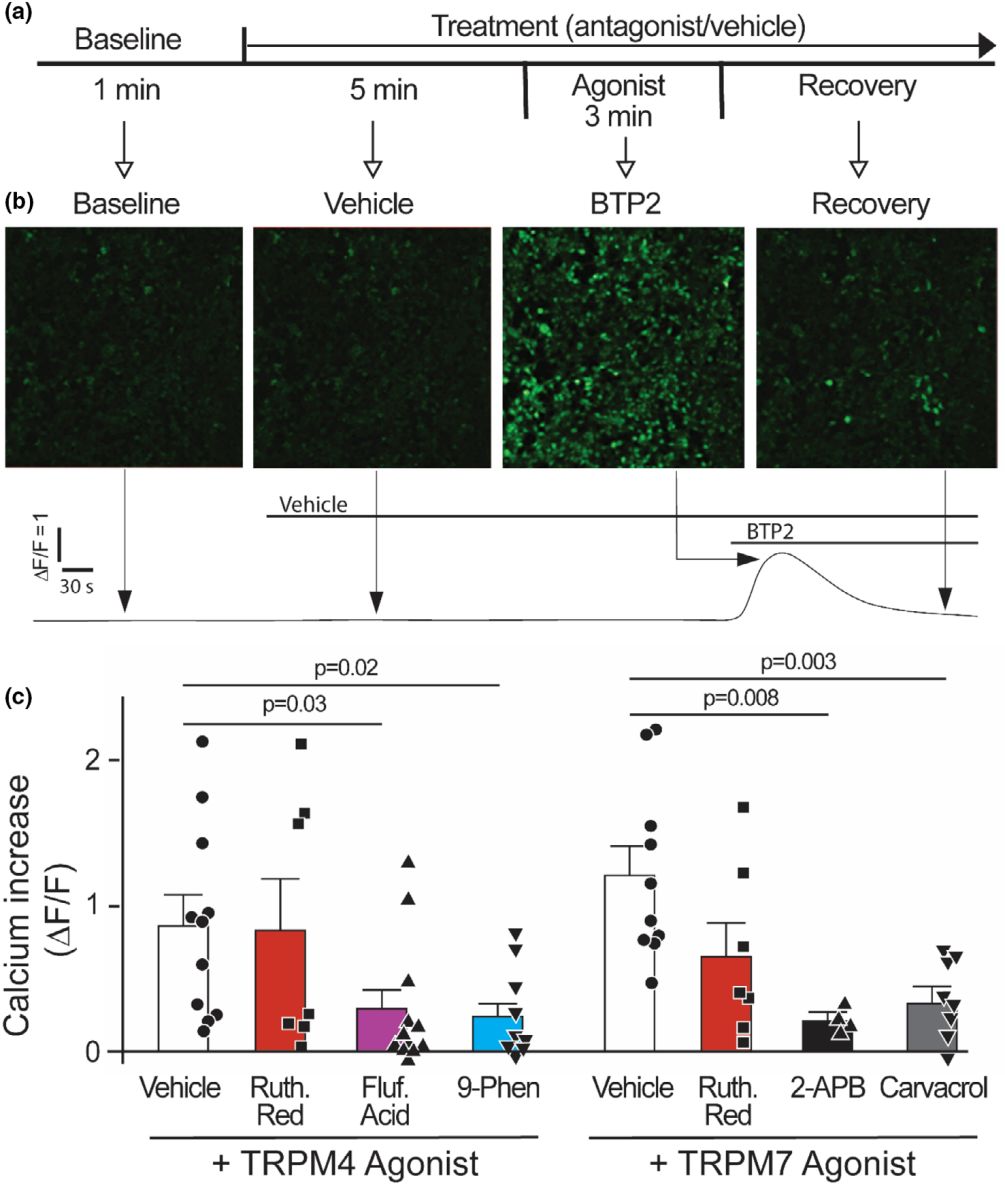
Pharmacological activation of TRPM4 or TRPM7 mobilizes Ca^2+^ in primary coronary pericytes. (a) Workflow for agonist induced Ca^2+^ mobilization in Fluo‐4 AM loaded cultures. AUC for a 1 min increment immediately following bath injection of TRPM4 agonist BTP2 or TRPM7 agonist Naltiben is compared to the AUC prior to any activity. (b) Representative readout of calcium fluorescence before, during, and after an agonist stimulation demonstrating a prominent increase in internal calcium fluorescence within seconds of agonist injections. (c, left panel) The TRPM4 agonist BTP2 (100 nM) induced strong Ca^2+^ mobilization when pre‐treated with vehicle alone (87.8 ± 19.9%, *n* = 11). Pretreatment with the TRP channel modulator Ruthenium Red had no significant impact on this effect (10 μM, 85.4 ± 33.1%, *p* = 0.99, *n* = 7), while pre‐incubation with TRPM4 antagonists Flufenamic acid (50 μM) and 9‐Phenanthrol (1 μM) significantly decreased this effect (29.8 ± 12.7%, *n* = 12; and 24.9 ± 8.5%, *n* = 11, respectively). (c, right panel) The TRMP7 agonist Naltriben (100 nM) also caused an increase in Ca^2+^ when pre‐treated with vehicle alone (122 ± 19.2%, *n* = 10). Pretreatment with Ruthenium Red caused no significant change to Naltriben (10 μM, 66.4 ± 22.7%, *p* = 0.02, *n* = 7). TRPM7 antagonists significantly decreased the Naltriben induced Ca^2+^ mobilization (2‐APB, 100 μM, 23.0 ± 7.9%, *n* = 4; Carvacol, 600 μM, 35.5 ± 9.6%, *n* = 8). All conditions were compared using Δ*F*/*F*.

As shown by us previously, real‐time, live‐cell confocal imaging of pericytes loaded with the cell permeant Ca^2+^ dye Fluo‐4 AM demonstrated clear Ca^2+^ mobilization in response to the canonical activator ATP (Methner et al., 2021). We next bath‐applied activators of either TRPM4 or TRPM7 to Fluo‐4 AM loaded cells (Figure 3a). For quantification, the area under the curve (AUC) for a 1 min increment during agonist application was compared as a percent change from the 1 min AUC baseline prior to any treatment (Δ*F*/*F*) (Figure 3b). Both the TRPM4 agonist BTP2 (also known as YM 58483; 100 nM) and the TRPM7 agonist Naltriben (100 nM) induced rapid Ca^2+^ mobilization. This effect persisted with pre‐incubation of the broad spectrum TRP channel modulator Ruthenium Red that does not block TRPM4 or TRPM7 channels (Figure 3c). Pre‐incubation with the nonspecific TRPM4 antagonist Flufenamic Acid or the highly specific TRPM4 antagonist 9‐Phenanthrol significantly decreased Ca^2+^ mobilization induced by BTP2 (Figure 3c, left panel). Similarly, pre‐incubation with the nonspecific TRPM7 antagonist 2‐aminoethoxydiphenyl borate (2‐APB) or the specific TRPM7 inhibitor Carvacrol significantly decreased Ca^2+^ mobilization induced by Naltriben (Figure 3c, right panel). Together, these data suggest that activation of TRPM4 or TRPM7 leads to Ca^2+^ mobilization in primary cardiac pericytes.

### 3.4 Compressive force induces rapid Ca^2+^ mobilization in pericytes through TRPM4 and TRPM7 channels

**FIGURE 4:**
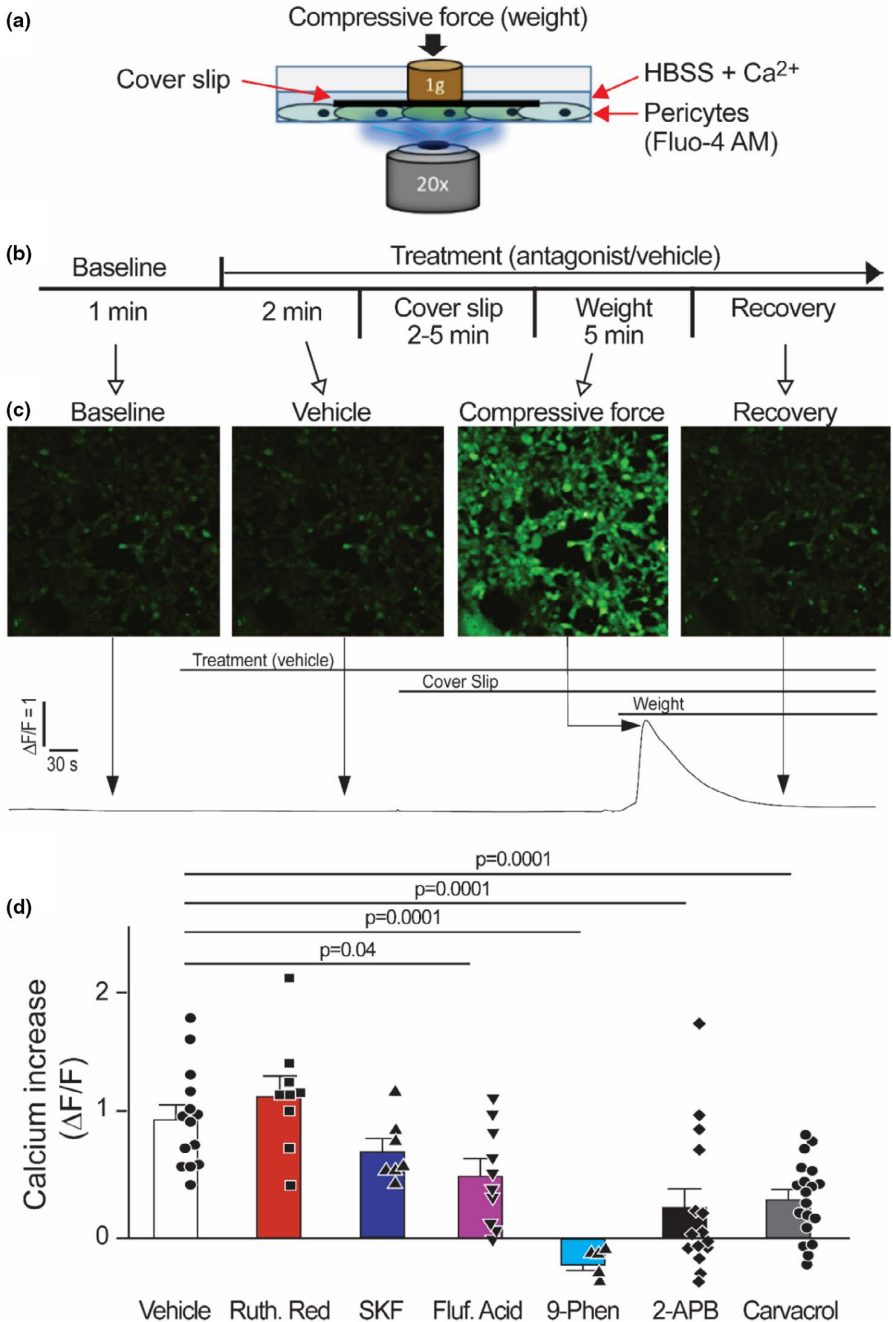
TRPM4 and TRPM7 mediate Ca^2+^ mobilization in primary cardiac pericytes induced by compressive force. (a) Depiction of compressive force application. Cultured primary glass pericytes in glass bottom dishes are loaded with Fluo‐4 AM. Then a thin glass coverslip is slid overtop to allow calibration weights to gently apply a compressive force without visible damage to cellular integrity. (b) Workflow for experimental series. Area under the curve (AUC) for 1 min immediately following compressive force is compared to a 1 min AUC of no activity. (c) Representative readout of Ca^2+^ fluorescence before, during, and after a compressive force experiment. Compressive force induces a clear Ca^2+^ mobilization without tearing or lifting cells. (d) Compressive force induces an immediate increase in Ca^2+^ mobilization in vehicle treated plates (Δ*F*/*F* = 100 ± 11%, *n* = 14) that is not significantly altered by pre‐incubation with Ruthenium Red (10 μM, 120 ± 15.9%, *n* = 9) or TRPC antagonist SKF96365 (10 μM, 73.5 ± 10.3%, *n* = 7). Ca^2+^ mobilization induced by compressive force was, however, significantly decreased by pre‐incubation with antagonists to TRPM4 (Flufenamic Acid: 50 μM, 53.3 ± 12.4%, *n* = 11; 9‐Phenanthrol: 3 μM, −19.9 ± 5.5% [Δ*F*/*F* decreased from baseline], *n* = 5) and TRPM7 (2‐APB: 50 μM, 26.6 ± 15.2%, *n* = 15; Carvacol: 400 μM, 33.1 ± 7.3%, *n* = 19). All conditions were compared using ANOVA.

To determine whether TRPM channels also mediate Ca^2+^ mobilization induced by pressure stimuli, cells were treated with antagonists to various TRP channels prior to compressive force application. The AUC for a 1 min increment during compressive force was compared as a percent change from the 1 min AUC prior to any activity (Figure 4a–c). The numerical results are depicted in Figure 4d. With vehicle, a strong Ca^2+^ signal immediately follows the compressive force. Neither Ruthenium Red nor the TRPC inhibitor, SKF96365, significantly changed compression‐induced Ca^2+^ mobilization. However, preincubation with TRPM4 antagonists Flufenamic Acid or 9‐Phenanthrol significantly decreased the compressive force induced Ca^2+^ mobilization. Similarly, preincubation with TRPM7 antagonists 2‐APB or Carvacrol significantly decreased Ca^2+^ mobilization induced by compressive force. All antagonist responses were normalized to their respective vehicle treatments (H_2_O, ETOH, or DMSO), which were not significantly different from naïve control experiments. Altogether, these data suggest that both TRPM4 and TRPM7 play a role in mobilizing Ca^2+^ following compressive force in primary cardiac pericytes.

### 3.5 TRPM4 inhibition reduces infarct size in vivo

**FIGURE 5:**
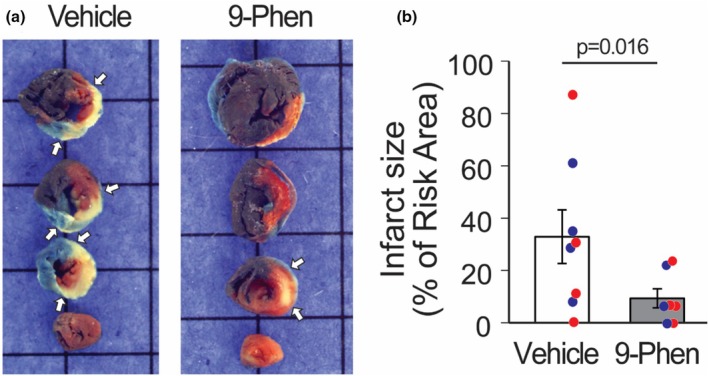
TRPM4 Blocker 9‐Phenanthrol markedly reduces infarct size in mouse model. Infarct size data from animals treated with vehicle (*n* = 8) and 9‐phenanthrol (*n* = 7). A large infarct (pale tissue unstained with TTC and marked by white arrows) is noted in the vehicle treated animal (a) while the infarct size in a 9‐phenanthrol treated animal is much smaller. The risk area is represented by tissue not stained with Evan's Blue. Aggregate data (b) shows a significant difference in infarct size/risk area ratio between the two groups of animals. There is a 3.5‐fold reduction in the infarct size/risk area ratio in 9‐phenanthrol treated compared to vehicle treated animals (9.4 ± 9.7% vs. 32.9 ± 28.9%). Red denotes females and blue denotes males.

Pericytes contract in response to increases in intracellular Ca^2+^ and cause capillary constriction, contributing to larger infarct size following AMI (Methner et al., 2021). Hence, we examined the role of TRPM4 in an in vivo AMI mouse model. Coronary occlusion followed by reperfusion resulted in a large infarct (pale tissue unstained within TTC) in vehicle‐treated animals (Figure 5a, left panel) whose risk area (tissue not stained with Evan's Blue) was similar in size to a 9‐phenanthrol‐treated animal. Yet, the infarct size was smaller in the latter group (Figure 5a, right panel). Aggregate data (Figure 5b) show a 3.5‐fold reduction in infarct volume in 9‐phenanthrol‐treated animals (9.4 ± 9.7%) compared to vehicle‐treated animals (32.9 ± 28.9%, *p* = 0.016). These data suggest that TRPM4 plays an important role in AMI, potentially by promoting pericyte contraction when intramyocardial pressure increases.

## 4 DISCUSSION

Using an in vitro culture system, we demonstrate that TRPM4 and TRPM7 channels are present in cardiac pericytes and, when activated pharmacologically, they mobilize intracellular Ca^2+^. Application of compressive force also mobilizes intracellular Ca^2+^ in pericytes through TRPM4 and TRPM7‐dependent mechanisms. Blocking TRPM4 after coronary occlusion in an in vivo rodent AMI model reduces the infarct size/risk area ratio by more than two‐thirds. A previous report showed that pre‐treatment with carvacol, a specific inhibitor of TRPM7, also reduces infarct size (Yu et al., 2013). Taken together, these results imply that TRPM channels in pericytes can mechanosense changes in intramyocardial pressure, likely inducing capillary constriction and contributing to ischemic tissue damage. Drugs that prevent TRPM channel activation may serve as an early treatment for AMI.

Brain pericytes possess a large number of ion channels and G‐protein coupled receptors to sense their microenvironment, and their mechanobiology has been well studied (Dessalles et al., 2021; Hariharan et al., 2020). Cardiac pericytes have not been as extensively studied, although they are the second most abundant cells in the heart (Nees et al., 2012). Recent studies have reported their in vitro characterization, including proteomics, response to pharmacologic agents, and secretome (Cao et al., 2022; Dalkara et al., 2024; Lee et al., 2021; Su et al., 2021). Insights into their role in cardiac microvascular fluid homeostasis are just beginning to emerge (Kaul et al., 2020, 2023; Le et al., 2022; Methner et al., 2019, 2021; O'Farrell et al., 2017).

We (Kaul et al., 2023; Methner et al., 2021; O'Farrell et al., 2017) and others (Dalkara et al., 2024) have previously shown that pericyte contraction during myocardial ischemia results in reduced capillary diameter and density, contributing to no reflow and larger infarct size. We reported that increased tissue levels of the vasoconstrictor 15‐hydroxyeicosatetraenoic acid (15‐HETE) during myocardial ischemia result in pericyte contraction through activation of the G‐protein coupled receptor 39 (GPR39) (Alkayed et al., 2022; Methner et al., 2021) that leads to an increase in pericyte cytosolic Ca^2+^ resulting in their contraction and capillary constriction (Methner et al., 2021). GPR39 knockout (KO) mice and mice treated with a specific GPR39 inhibitor have marked reductions in infarct size (Methner et al., 2021). Our current results showing a role for TRPM4 in cardiac pericyte Ca^2+^ mobilization and infarct formation suggest that multiple pericyte‐dependent pathways may contribute to AMI.

Unlike the eye and brain, the two organs where pericytes have been most studied, cardiac pericytes are exposed not only to intraluminal pressure within the capillaries, but also to cyclic fluctuations in the surrounding intramyocardial pressure. Like the brain and eye, they are also exposed to biomolecules that are associated with different physiological and pathological conditions. For instance, when coronary perfusion pressure drops below normal resulting in ischemia, adenosine, endothelin, and 15‐HETE, among other chemicals, are released. And while adenosine can relax cardiac pericytes (Alkayed et al., 2022; O'Farrell et al., 2017), they actually contract and constrict capillaries (Aversano & Becker, 1985; Canty & Klocke, 1985; Jayaweera et al., 1999; Kaul et al., 2023; Le et al., 2004, 2024; Methner et al., 2021) from the effect of 15‐HETE and other substances causing microvascular resistance to increase (Aversano & Becker, 1985; Jayaweera et al., 1999; Le et al., 2004). This increased resistance can be overcome by pharmacological doses of adenosine (Aversano & Becker, 1985; Canty & Klocke, 1985; O'Farrell et al., 2017) and GRR39 inhibitors that relax the contracted pericytes (Kaul et al., 2023; Le et al., 2024; Methner et al., 2021).

We have discussed the role of cardiac pericytes in tissue fluid homeostasis before (Kaul et al., 2020, 2023; Le et al., 2022; Methner et al., 2019, 2021) and shown that it may be simply related to alterations in intraluminal perfusion pressure. When perfusion pressure falls, pericytes contract to maintain constant capillary hydrostatic pressure, one of the four components of Starling's forces, the other three being capillary oncotic pressure and interstitial hydrostatic and oncotic pressures, respectively (Starling, 1896). It is these four forces that maintain tissue fluid homeostasis at the capillary level. Therefore, sensing any potential changes in capillary hydrostatic pressure and responding immediately requires mechanosensing either in the endothelium or the contractile cells lining them.

Myocardial pericytes are likely to undergo some deformation during cardiac contraction that becomes exaggerated during ischemia where the intramyocardial pressures not only increase, but the normal pattern of contraction gets out of phase, which may result in a pronounced push‐pull effect on the pericytes. As we have shown in the current study, mechanosensors present in cardiac pericytes (TRPM4 and TRPM7, and there are probably others [Earley, 2013; Inoue et al., 2009; Starostina et al., 2021]) sense this distortion causing in increased cytosolic Ca^2+^ and pericyte contraction. Thus, capillary constriction results in a situation where capillary flow is already reduced, worsening the ischemia. A previous report shows that pre‐treatment with carvacol, a TRPM7 inhibitor, results in smaller infarct size (Yu et al., 2013). Our results indicate that inhibiting TRPM4 even after coronary occlusion results in a reduction in infarct size, probably by preventing pericyte contraction.

From a therapeutic perspective, it may be beneficial to address pericyte function in myocardial ischemia using a multipronged approach. For example, it has been previously shown that adenosine relaxes pericytes and increases capillary flow in AMI (O'Farrell et al., 2017). Our previous work showed that inhibition or deletion of GPR39 reduces infarct size (Methner et al., 2021), and our current results show infarct size reduction by altering abnormal mechanotransduction through TRPM4 inhibition. Reduction of oxidative stress is presumed to be another mechanism by which TRPM7 inhibition could reduce infarct size (Yu et al., 2013). Thus, a combinatorial approach to prevent pericyte contraction from different perspectives (vasodilatory, mechanosensory, oxidative stress, etc.) may offer maximal benefit by addressing the maladaptation of the microcirculation to ischemia. This would be in addition to removing the proximate cause of myocardial ischemia, such as an occlusive thrombus in an epicardial artery.

Cardiac pericytes play other important roles in AMI. Isolated human cardiac pericytes promote angiogenesis and network formation, with potential increases in tissue perfusion and healing (Chen et al., 2015). In vivo, a subset of cardiac pericytes also seems to undergo transformation into cardiomyocytes when the myocardium is exposed to 5‐azacytidine (Chen et al., 2015). In heart failure, pericytes lose mechanotransduction properties, which could be reversed pharmacologically (Rolle et al., 2021). Thus, pericytes may be critical yet underappreciated players in alleviating tissue damage after myocardial ischemia or other heart conditions.

Although we have only examined AMI in this study, intramyocardial compressive forces are elevated in other conditions as well, such as hypertension, aortic stenosis, and hypertrophic cardiomyopathy, all of which have reduced capillary density (Güçlü et al., 2015; McConkey et al., 2019; Rakusan et al., 1992; Tomanek et al., 1982), a putative cause for which could be pericyte contraction. Reduced capillary density results in decreased coronary blood flow reserve (Jayaweera et al., 1999) and myocardial ischemia in these conditions, further worsening their outcomes. Thus, inhibiting TRPM4 may limit pericyte contraction and enhance microvascular flow in such pathologies.

### 4.1 Study limitations

The use of cultured pericytes is a limitation of our study. However, several pieces of evidence support that our findings are applicable in vivo also. Different types of TRP channels are expressed in capillary pericytes (Cao et al., 2022; Hariharan et al., 2020) and TRPM4 has been shown to mediate pericyte contraction in other organ systems, such as the retina (Gonzales & Nelson, 2014). That TRPM4 inhibition strongly reduced infarct size after AMI in vivo further curbs this concern. Future studies are needed to definitively test whether this effect of TRPM4 inhibition is due to improved capillary flow. Further, a dose–response of 9‐phen for limitation of infarct size needs to be established. Studies are also needed to combine the inhibition of various TRPM channels to determine optimal combination doses for therapeutic translation. Finally, TRPM4 inhibition may be beneficial to the ischemic myocardium via mechanisms other than increasing blood flow. For instance, TRPM channels have been implicated in oxidative stress signaling and apoptosis, which obviously is active in AMI (Jiang et al., 2023; Yu et al., 2013).

## 5 CONCLUSIONS

Pericytes sense increased compressive forces (pressure) via mechanosensors such as TRP channels. Myocardial ischemia results in abnormal regional function with increased intramyocardial compressive forces that are sensed by pericytes, which then respond by contracting, thus resulting in capillary constriction and additional ischemia. Inhibiting TRP channels reduces infarct size following AMI and may offer a therapeutic option in patients experiencing AMI.

## AUTHOR CONTRIBUTIONS

EC, JI, and ZC designed and executed the in vitro experiments. SK and CM designed and executed the in vivo experiments. AM assisted in data analysis and figure preparations of the in vitro experiments. EC, AM, and SK drafted the first version of the manuscript. CM, AM, and SK edited the manuscript.

## FUNDING INFORMATION

Eugene Cilento's work was supported, in part, by a T‐32 Fellowship grant (HL‐09429) from the National Heart, Lung, and Blood Institute, National Institutes of Health, Bethesda, MD, USA. The Research in this paper was funded in part by the Garthe and Grace L. Brown and the John and Robin Jaqua Funds at the Oregon Community Foundation, Portland, OR, USA.

## CONFLICT OF INTEREST STATEMENT

No conflicts of interest, financial or otherwise, are declared by the authors.

## ETHICS STATEMENT

Studies adhered to NIH guidelines for research in animals.

## Data Availability

All experimental data presented in this study were collected specifically for this manuscript and have not been published in any previous works from this laboratory or any other. The data that support the findings of this study are available from the corresponding author upon reasonable request.
